# Investigating the Effects of COVID-19 Quarantine in Migraine: An Observational Cross-Sectional Study From the Italian National Headache Registry (RICe)

**DOI:** 10.3389/fneur.2020.597881

**Published:** 2020-11-10

**Authors:** Marianna Delussi, Eleonora Gentile, Gianluca Coppola, Addolorata Maria Pia Prudenzano, Innocenzo Rainero, Grazia Sances, Chiara Abagnale, Valeria Caponnetto, Francesco De Cesaris, Ilaria Frattale, Elena Guaschino, Andrea Marcinnò, Raffaele Ornello, Francesca Pistoia, Alessia Putortì, Maria Elena Roca, Fausto Roveta, Chiara Lupi, Maria Trojano, Francesco Pierelli, Pierangelo Geppetti, Simona Sacco, Marina de Tommaso

**Affiliations:** ^1^Applied Neurophysiology and Pain Unit, Scienze Mediche di Base, Neuroscienze e Organi di Senso Department, Bari Aldo Moro University, Bari, Italy; ^2^Sapienza University of Rome Polo Pontino, Latina, Italy; ^3^Headache Center, Amaducci Neurological Clinic, Policlinico General Hospital, Bari, Italy; ^4^Department of Neuroscience “Rita Levi Montalcini,” Headache Center, University of Torino, Turin, Italy; ^5^Headache Science Centre, Istituto di Ricerca a Carattere Scientifico Mondino Foundation, Pavia, Italy; ^6^Headache Center, Sapienza Rome University, Latina, Italy; ^7^Headache Regional Referral Center, Azienda Sanitaria Locale 1 Abruzzo, L'Aquila University, L'Aquila, Italy; ^8^Headache Center, Careggi General Hospital, Firenze, Italy; ^9^University of Florence, Florence, Italy; ^10^Sapienza University of Rome Polo Pontino, Latina, IRCCS - Neuromed, Pozzilli, Italy

**Keywords:** migraine, COVID-19, lockdown, resilience, disgust

## Abstract

**Background:** Previous studies during SARS and Ebola pandemics have shown that quarantine is associated with several negative psychological effects, such as post-traumatic stress symptoms, confusion, and anger. These conditions may affect the course of many diseases, including migraine. Although it is possible that the quarantine measures for the current COVID-19 pandemic affect migraine burden, no information is currently available on this issue.

**Aim:** In this study, we aimed to: (1) explore the possible changes in migraine frequency, severity, and days with acute medication intake during quarantine period; (2) evaluate possible differences in migraine outcomes in consideration of lifestyle changes, emotions, pandemic diffusion, and COVID-19 infection.

**Methods:** We interviewed patients who were included in the observational Italian Headache Registry (Registro Italiano Cefalee, RICE), retrospectively collecting information on main headache features, lifestyle factors, emotions, individual infection status, and perception of COVID-19 for 2 months before (pre-quarantine) and after the beginning of the quarantine (quarantine). Inclusion criteria were: age > 18, diagnosis of migraine without aura, migraine with aura and chronic migraine, last in-person visit more than 3 months preceding the beginning of quarantine.

**Results:** A total of 433 migraine subjects agreed to be interviewed. We found an overall reduction in headache frequency (9.42 ± 0.43 days with headache vs. 8.28 ± 0.41) and intensity (6.57 ± 0.19 vs. 6.59 ± 0.21) during the quarantine, compared to pre-quarantine. There was a correlation between improvement and number of days of stay-at-home. When results were stratified for geographic area, we found a tendency toward worsening of headache frequency in northern Italy. Disgust regarding viral infection corresponded to a minor improvement in migraine.

**Conclusions:** Migraine patients showed a mild improvement of migraine features, probably attributable to resilient behavior toward pandemic distress. Disgust regarding the contagion whereas potentially favoring defensive behavior, could potentially worsen migraine. The spontaneous limitation of migraine burden during quarantine could favor patient follow-up via the use of telemedicine visits, reliable diaries, and frequent remote contacts.

## Introduction

COVID-19 (1, 2) was declared a global pandemic on March 11, 2020 by the World Health Organization. Italy, the first European country in which there was an outbreak of the pandemic, currently records a total of ~185,000 confirmed cases and more than 24,000 patients with severe illness. The spread of COVID-19 in the Italian territory was markedly different, with northern Italy showing a much higher number of cases compared to central and southern Italy. On March 10, the Italian government was the first in Europe to impose severe social-distancing orders. Social-distancing and mitigation strategies (3–5) aim to defer a major flow of patients and reduce the demand for hospital admissions while safeguarding the most vulnerable subjects (6).

Studies related to the 2003 outbreak of severe acute respiratory syndrome (SARS) in China and Canada, as well as the 2014 Ebola outbreak in Africa, reported that quarantine is associated with several negative psychological effects, such as post-traumatic stress symptoms, confusion, and anger (7, 8), with a potential increase in suicidal risk (9). Changes in social behavior and work activities, the unavailability of a public health system for routine medical management, and widespread fear of infection could cause important psychosocial outcomes (10–13), and could dramatically increase the burden of the disease. However, few data have been reported on the impact on migraine caused by psychosocial distress due to COVID-19 and long-term distancing measures.

In this study, we aimed to (1) explore whether the quarantine period affected the frequency and severity of migraine and days with acute medication intake, and (2) evaluate possible differences in migraine outcomes in consideration of changes in lifestyle, emotions, pandemic diffusion, and COVID-19 infection.

## Methods

### Study Population and Design

This observational cross-sectional study describes the impact of the COVID-19 pandemic and social distancing measures on headache features in migraine patients. It was conducted via a structured telephone interview in a sample of patients included in the Italian Registry of Headache (Registro Italiano delle Cefalee, RICe), which enrolls patients aged ≥ 18 years who visit headache treatment centers.

RICe is an observational registry promoted and endorsed by the Italian Society for the Study of headaches (SISC), which records clinical data of consecutive patients with headache who refer to the member Headache Centers. The present ancillary sub-study is based on data from seven headache centers in northern (Pavia and Turin), central (Florence and Latina), and southern (Avezzano-L'Aquila, and two centers in Bari) that enrolled patients who had entered the RICe at least 3 months before the start of the quarantine (pre-quarantine) in Italy. The seven centers are located in geographical areas that experienced markedly different impacts of the COVID-19 outbreak: higher in the north, medium central, and lower in southern Italy (2020- http://www.protezionecivile.gov.it/attivita-rischi/rischio-sanitario/emergenze/coronavirus). All the patients admitted to the RICe study were requested to complete a headache diary, reporting the occurrence and intensity of headache (from 1 -mild to 10- the most severe headache), and use of symptomatic drugs. For the present study, the inclusion criteria were age ≥ 18 years, a diagnosis of migraine without aura, migraine with aura and chronic migraine according to the criteria of the International Classification of Headache Disorders, III edition (ICHD-III) (14), and the most recent in-person visit within the 3 months preceding the lockdown period. Exclusion criteria were ascertained comorbidity with other forms of primary headaches, psychiatric disorders according to DSMV, and liver, kidney, and heart insufficiency.

### Telephone Interviews and Variables of Interest

Telephone interviews were carried out by study investigators between March 27 and April 18, 2020. The interview was a web-supported questionnaire to be completed during the telephone call. The questionnaire was administered in the Italian language (it is available in Italian and English in the Supplementary Material). Variables of interest included frequency of headache expressed as average number of headache days per month, calculated during the 2 months preceding the quarantine (pre-quarantine) and in the time from the beginning of the quarantine (March 8, 2020 for Northern Italy, extended on March 10, 2020 to the rest of Italy). Patients were asked to report the intensity of headache and the use of symptomatic drugs during the pre-quarantine and quarantine times, according to their headache diaries. Questions included the following: number of days of staying at home, current working conditions, level of risk contacts, individual infection, personal feelings on how COVID-19 affects migraine outcomes and/or migraine as a possible risk factor for COVID-19, fear of becoming infected, possible changes in daily behaviors (food intake habits, alcohol consumption, and sleep quality) as a result of social-distancing measures, main sensations regarding the pandemic emergency (anger, fear, disgust, anxiety, sadness, happiness) on a scale from 0 (no emotion) to 10 (maximum emotion), and subjective evaluation of mood change (worsening, no change, improvement) (see the questionnaire in the Supplementary Section). The questionnaire was developed taking into consideration the headache features reported in the diaries. For questions related to the pandemic, we used *ad-hoc* and not previously validated scales on the possible changes of fundamental emotions, psychological reactions, and habits due to pandemic. A similar survey was used on the general Italian population during the current pandemic [(15); https://www.cnr.it/it/news/9363/risultati-dell-osservatorio-sui-mutamenti-sociali-in-atto-covid19-msa-covid19].

### Study Outcomes

Study outcomes were headache frequency and intensity and days with acute medication intake during the 8 weeks preceding and during the social-distancing measures. Predictor variables were migraine severity before the quarantine, lifestyle habits, emotions, severity of pandemic diffusion, and COVID-19 infection.

### Ethics

The local ethics committees of each recruiting center approved the RICe registry, and patients enrolled signed an informed consent, which included the possibility to perform sub-studies on their headache features.

### Statistical Analysis

In the absence of previous similar reports and because of the descriptive nature of the study, the sample size was not calculated. The parametric distribution of the data was evaluated by the Levene test for equality of variance. As the observation period during quarantine varied across subjects, we normalized headache days for the effective period of observation (number of headache days / number of total days of observation ×30). We used ANOVA for repeated measures with condition before vs. during social distancing as factors. The effect of severity of migraine before quarantine, emotions, living behavior, and geographic area on primary outcomes was evaluated by the same repeated measures ANOVA model, introducing nominal variables as factors and quantitative variables as covariates. A complete factorial ANOVA model type III included in IBM SPSS software version 21 was used.

## Results

### Demographic Data, Working Status, Living Conditions, and Daily Habits

#### General

A total of 433 migraine subjects agreed to be interviewed, while 10 patients did not give their consent. The mean interval elapsed between the start of the quarantine and the time of the interview was 31.9 ± 4.5 days without significant differences across participating centers. The baseline characteristics of the enrolled patients are presented in Table 1.

**Table 1:** Baseline characteristics of the included subjects.

	***n* = 433**
Gender, *n* (%)	
Female	333 (76.9)
Male	100 (23.1)
Age (years), mean ± SE	43.97 ± 0.63
BMI, mean ± SE	24.1 ± 0.002
Education years, *n* (%)	
0–5	16 (3.7)
6–8	84 (19.4)
9–13	175 (40.4)
>13	158 (36.5)
Days of social distancing, mean ± SE	29.27 ± 0.58
Home place, *n* (%)	
Countryside	76 (17.8)
City	183 (42.3)
Small town	174 (40.2)
No. of cohabiting family members during social distancing, mean ± SE	2.32 ± 0.13
Work, *n* (%)	
Unemployed	208 (48.0)
Employed	225 (52.0)
Employment, *n* (%)	
Work from home	89 (20.6)
At workplace	48 (11.1)
Unemployed (lost position)	88 (20.3)
Food intake, *n* (%)	
Increased	67 (15.5)
Reduced	153 (35.3)
Unchanged	213 (49.2)
Alcohol consumption, *n* (%)	
Reduced	85 (19.6)
Increased	13 (3.0)
Unchanged	344 (79.4)
Sleep quality, *n* (%)	
Improved	157 (36.3)
Worsened	45 (10.4)
Unchanged	229 (52.9)
Emotional reaction, mean ± SE	
Anger	4.15 ± 0.27
Disgust	3.39 ± 0.27
Fear	5.71 ± 0.28
Anxiety	5.86 ± 0.18
Sadness	5.40 ± 0.28
Happiness	4.67 ± 0.23

*BMI indicates body mass index*.

*Intensity of emotions related to the COVID-19 emergency are reported on a 0–10 scale*.

Most of the interviewed patients did not report changes in food intake, sleep, or alcohol consumption (Table 1). During quarantine, 177 subjects (55.1%) reported worsening, 42 (13.1%) no change, and 102 (31.8%) improvement in mood. Emotions scores related to pandemic are reported in Table 1.

#### Migraine During Quarantine

A reduction in the average number of days with headache (9.42 ± 0.43 before quarantine, 8.28 ± 0.41 during quarantine), days with acute medication intake (10.21 ± 0.96 before; 10.79 ± 1.02 during), and migraine intensity (6.57 ± 0.19 before; 6.59 ± 0.21 during) was observed during quarantine compared to pre-quarantine (Table 2). Most patients subjectively reported that their migraines did not change after the start of quarantine. Most migraine patients did not consider migraine as a facilitating factor for COVID-19 infection (Supplemental Table 1). Two hundred eighty-nine patients were taking preventive treatments for migraine; 88 discontinued treatment for different reasons, such as drug failure or difficulty in attaining the drugs (Supplemental Table 2).

**Table 2:** Comparison of headache features before and during lockdown period.

**n^**°**^ 433**	**Before**	**During**	***F***	***P*-value**	**EM before n^**°**^331**	**During**	**CM before n^**°**^102**	**During**	***F***	***P*-value**
Monthly headache days	9.42 ± 0.43	8.28 ± 0.41	60.6	<0.0001	5.10 ± 0.23	4.99 ± 0.35	21.6 ± 0.39	16.74 ± 0.59	59.6	<0.0001
Acute medication days	8.32 ± 0.51	7.19 ± 0.54	21.7	<0.0001	4.91 ± 0.46	4.2 ± 0.55	16.9 ± 0.78	13.2 ± 0.93	13.76	<0.0001
Headache intensity	6.93 ± 0.10	6.71 ± 0.11	6	0.014	6.79 ± 0.11	6.5 ± 0.12	7.25 ± 0.25	6.95 ± 0.19	0.1	n.s.

*CM, Chronic Migraine patients; EM, Episodic Migraine patients. Data are reported as mean ± standard errors*.

#### Effects of Migraine Severity, Lifestyle Habits, Emotions, and Severity of Pandemic Diffusion on Headache Frequency

**Figure 1:**
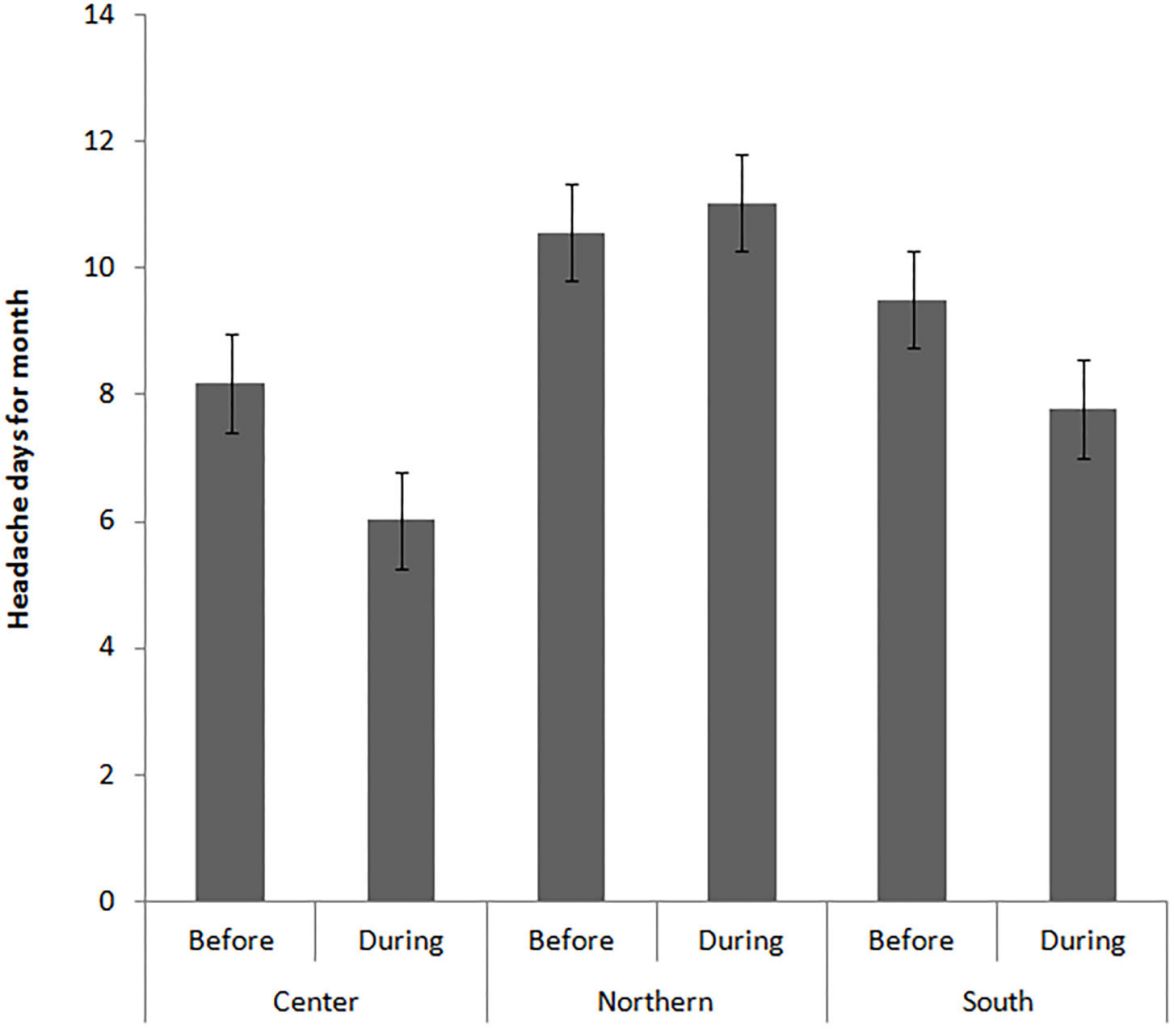
Mean and standard errors of headache frequency, expressed in number of headache days for month, before and during the quarantine in the different Italian geographic areas. Statistical analysis is reported in Table 3.

Before quarantine, 331 patients reported episodic migraine, while the remainder were affected by chronic migraine. Improvements in headache frequency and analgesic consumption were higher in chronic patients than in episodic migraine patients (Table 2). Alcohol use, smoking, eating, and subjective perception of sleep quality did not affect headache frequency and intensity, use of symptomatic drugs, and/or working conditions. The improvement of headache frequency correlated with the number of stay-at-home days (ANOVA with repeated measures with days of social distancing as covariate *F* 37.07 *p* < 0.0001). Changes in headache parameters were similar among patients living in different urban areas and with different levels of education. Migraine features were not significantly different among northern, central and southern Italy before the quarantine period (Supplemental Table 3). However, patients in northern Italy, the geographical area with the highest pandemic diffusion, showed a tendency of increased headache frequency and use of acute medication (Table 3; Figure 1). It should be noted that the number of days of effective stay-at-home was reduced in patients from northern Italy (Supplemental Table 3).

**Table 3:** Comparison of headache features before and during lockdown period in the included patients.

	**North (*****n*** **=** **105)**		**Center (*****n*** **=** **101)**		**South (*****n*** **=** **227)**		***F* (geographic area)**	***P*-value**	***P*-value (Bonferroni)**
	**Before**	**During**	**Before**	**During**	**Before**	**During**			
Monthly headache days	10.57 ± 0.81	11.03 ± 0.78	8.19 ± 0.84	6.02 ± 0.80	9.50 ± 0.56	7.78 ± 0.53	6	0.003	<0.001 (South and Center vs. North)
Acute medication days	10.21 ± 0.96	10.79 ± 1.02	7.29 ± 0.98	5.07 ± 1.04	7.46 ± 0.65	5.70 ± 0.69	3.6	0.027	<0.01 (South and Center vs. North)
Headache intensity	6.57 ± 0.19	6.59 ± 0.21	7.25 ± 0.21	7.03 ± 0.22	6.96 ± 0.21	6.50 ± 0.14	3	0.05	NS

*Data are reported as mean ± standard errors*.

There was a significant relationship between disgust against COVID-19 infection and an increase in headache frequency (repeated measures ANOVA with disgust as covariate *F* 6.43 *p* 0.004).

Patients reporting mood improvement showed a tendency toward reduced headache frequency (repeated measures ANOVA with mood perception as factor: *F* 5.43 *p* 0.001). However, the Bonferroni test among the different mood perceptions did not show any significant difference. Patients who subjectively reported a worsening of their migraine showed an objective increase in headache frequency (repeated measures ANOVA with subjective impression of migraine severity as factor: *F* 35.58 *p* < 0.0001: Bonferroni test: got worse vs. improved *p* < 0.01). However, patients feeling migraine as a facilitating factor for infection showed a tendency toward frequency increase (ANOVA with perception of migraine as risk factor: *F* 3.59 *p* 0.012 Bonferroni test: n.s.). Emotions against pandemic and subjective perception of mood and disease severity did not influence the change in headache features in chronic migraine compared to episodic migraine.

#### COVID-19 Infection in Migraine Samples

In the overall sample, five patients reported having been infected by SARS-CoV-2, two patients were asymptomatic, one had recovered, and two were under treatment for mild pulmonary symptoms that did not require hospitalization (Table 4). These patients did not report substantial changes in their migraines after infection.

**Table 4:** Characteristics of migraine patients positive to SARS-CoV-2 infection.

**Patient**	**Age**	**Sex**	**Geographic area**	**Symptomatic**	**COVID-19 Outcome**	**Migraine frequency change (%)**	**Mean intensity change (points)**	**Change in use of symptomatic drugs (%)**
1	22	F	South	No		−18	0.38	−75
2	56	F	North	No		−20	1	0
3	47	F	South	Yes	Under treatment	−20	0.88	0
4	44	F	North	Yes	Recovered	−40	1	−32
5	46	F	North	Yes	Under treatment	−30	0.5	0

*Changes refer to quarantine measures compared with the 2 previous months*.

## Discussion

The results of the present study show that migraine patients had a mild improvement of headache frequency, days with acute medication intake, and headache intensity during the social distancing measures. Migraine improvement prevailed in chronic patients in the areas where there was the lowest prevalence of COVID-19, and was positively influenced by the number of days of stay-at-home orders. Disgust toward infection corresponded to an attenuation of headache improvement.

### Migraine During Quarantine

The interview was conducted on migraine patients previously enrolled in RICe, which guaranteed the presence of accurate clinical features previously recorded. This represents a point of strength compared to interviews administered to the general population (16). The general improvement of headache frequency and intensity during lockdown confirms results obtained in smaller Italian migraine samples (17). Viral diffusion may contribute to the onset of stress-related disorders (12, 18), which may worsen migraine (19). The development of a resilient behavior associated with COVID-19 (18) may have also involved migraine patients. The American Psychological Association (2014) defines resilience as “the process of adapting well in the face of adversity, trauma, tragedy, threats, or even significant sources of stress.” During a pandemic, resilient behavior could reduce psychological distress (20). Although we did not apply a specific questionnaire for resilience, a resilient mechanism could help to improve the main features of migraine, such as frequency in terms of days of headache per month and subjective feelings of its intensity. In fact, resilience ability is generally associated with better outcomes in patients with chronic pain (21).

### Effects of Migraine Severity, Lifestyle Habits, Emotions, and Severity of Pandemic Diffusion on Headache Frequency

In a previous large global cross-sectional study, patients with frequent migraine showed greater resilience in response to negative events, such as treatment failures (22). The present study suggests that mild improvement of migraine, probably attributable to a resilient behavior against the pandemic, could prevail in more severe migraines. Psychological features of migraine patients, underlying a favorable outcome under the current dramatic epidemics, were not recorded in the RICe database upon previous visits, Although this could be subject to further studies in a possible scenario of pandemic persistence (23). A correlation between the emotional impact of the pandemic situation and its effect on migraine features was not found. However, the perception that migraine could facilitate COVID-19 infection, possibly causing additional stress, negatively affected the improvement of headache frequency. The pandemic emergency did not seem to cause particular sleep disruption in our migraine sample, an effect attributable to the resilient reaction.

The improvement of headache severity was correlated with the effective number of days of stay at home (24, 25). The resilient behavior against migraine worsening for pandemic distress could thus be enhanced during effective social distancing. We did not find an association between headache improvement, work activity, and lifestyle habits. Nevertheless, staying at home, even if forced, could globally influence trigger factors and the ability to rest, possibly decreasing the risk of recurrence. Environmental factors could cause physical and psychological distress in normal daily living, which would be attenuated during the quarantine. The usual pace of life for work, exposure to weather changes, car traffic, and travel, are recognized migraine stressors, and these were obviously reduced in this unusual condition (26). This result could be taken into consideration for the social management of public health during pandemics (5).

The headache centers cooperating in data collection see patients coming from different Italian regions, so we considered the place of residence at the time of public restrictive measures. Residents in regions with higher pandemic diffusion seemed to express less resilient behavior. As an adjunctive result, we also observed that patients in northern Italy reported fewer days of social distancing, which were associated with a positive outcome of migraine. We could thus suppose that the severity of pandemic diffusion could change environmental situations and personal habits (15) and exert an influence on resilient behavior.

The expression of disgust was slightly associated with migraine frequency increase. Disgust is an emotional response of rejection or revulsion to something potentially contagious or offensive (27). It is a system that evolved to motivate infectious disease avoidance and combat the behavioral causes of infectious and chronic diseases, such as pandemic flu (28). While it could help in assessing avoidance behavior during pandemic infections, it is a cause of distress (28), which could have a negative impact on migraine frequency and attenuate the resilient reaction.

### COVID-19 Infection in Migraine Samples

Although the present study was not designed to assess the frequency of infection in the migraine population, because of the relatively small sample, we did find 5 out of 433 migraine patients positive for SARS-CoV-2 infection (1.15%). At the time we collected data, there were ~185,000 infected individuals in the general Italian population (0.3%), with a slight prevalence in males (51.7%) and a median age of 62 years (Italian Healthy System and Civil Protection report http://www.protezionecivile.gov.it/attivita-rischi/rischio-sanitario/emergenze/coronavirus). However, the percentage of asymptomatic persons among the general population is an unresolved issue, so the present data do not enable any type of speculation about the prevalence of SARS-CoV-2 infection in migraine patients. Recent reviews about the main symptoms of COVID-19 stated that headache occurs in nearly 10% of patients, while migraine is not included within the comorbidities that aggravate symptomatic patients (29). The three patients presenting with symptoms of COVID-19 had a mild form, which did not require hospitalization. They experienced the same small improvement of headache symptoms as most of the other patients. In the current literature, data on migraine patients presenting with more severe symptoms of infection are still lacking. Further studies and meta-analyses are needed to establish the prevalence and clinical aspects of COVID-19 in the migraine population.

### Study Limitations

The main limitation of the present study is the small number of patients interviewed. This limitation was determined by the restricted time window of the interview, limited to the time of the restrictive measures, also termed “phase I,” enforced by the Italian Government, and by the fact that enrolled cases were solely those patients who had previously given their informed consent to the RICe study and therefore could be enrolled in the present sub-study. Additional limitations could be the scarce reliability of headache diaries during the pandemic and the short amount of time for evaluation of migraine outcome during the social and health emergency (1 month on average). In fact, the consistency of a retrospective report of headache frequency and patient habits by telephone interview could be questionable. This study was conducted in tertiary headache centers and included patients with high monthly attack frequency who were not representative of migraine in the general population. Finally, the interview text and scales for emotional and psychological impact of pandemic, were only partially validated among Italian citizens (15).

## Conclusions

We found that, on average, migraine patients expressed a reduction in migraine severity indices, probably due to resilient behavior with regards to pandemic distress. The maintenance of habitual lifestyles during social distancing was less evident in people with a limited number of days of staying at home. The present data could help in the future reorganization of services, healthcare workforce, and ongoing management of migraine (30). The spontaneous limitation of migraine burden during quarantine could favor patient follow-up *via* the use of telemedicine visits (12, 31), reliable diaries, and frequent remote contacts after an initial in-person visit.

## Data Availability Statement

The raw data supporting the conclusions of this article will be made available by the authors, without undue reservation.

## Ethics Statement

The studies involving human participants were reviewed and approved by Local Ethic Committees of each recruiting center approved the RICe registry, and enrolled patients signed informed consent. The patients/participants provided their written informed consent to participate in this study.

## Author Contributions

MD and EG: equally contributed, study design, interview preparation, patient selection, and study coordination. MdT: study coordination, manuscript preparation, data analysis, and manuscript editing. PG and SS: manuscript preparation, data analysis, and manuscript editing. IR, GC, GS, and AP: manuscript preparation and editing. FPie and MT: study design and manuscript editing. RO: study design and statistical analysis. CA, VC, FD, IF, EG, AM, FPis, AP, MR, and FR: patient selection, manuscript editing, preparation, and telephone interviews. CL: RICe data, patient selection, and telephone interviews. All authors contributed to the article and approved the submitted version.

## Conflict of Interest

The authors declare that the research was conducted in the absence of any commercial or financial relationships that could be construed as a potential conflict of interest.

## Abbreviations

RICe: Registro Italiano Cefalee- Italian Headache Registry
SISC: Società
Italiana Studio Cefalee-Italian Society for Headache Study.
