# Supplementary material for: Investigating the Effects of COVID-19 Quarantine in Migraine: An Observational Cross-Sectional Study From the Italian National Headache Registry (RICe)
Source: Front Neurol. 2020 Nov 10;11:597881. doi: 10.3389/fneur.2020.597881 (PMC7683429; doi:10.3389/fneur.2020.597881)
Supplement: Supplementary file 1 [file Data_Sheet_1.DOCX]

**APPENDIX A**

| Question | Answer |
| --- | --- |
| Gender | - Female - Male |
| Age | Open answer |
| Years of study | - 5 - 8 - 13 - > 13 |
| Weight | Open answer |
| Height | Open answer |
| Region of residence | - Abruzzo - Basilicata - Calabria - Campania - Emilia-Romagna - Friuli-Venezia Giulia - Lazio - Liguria - Lombardia - Marche - Molise - Piemonte - Puglia - Sardegna - Sicilia - Toscana - Trentino-Alto Adige - Umbria - Valle d'Aosta - Veneto |
| In which province do you currently live? | Open answer |
| In which municipality do you currently live? | Open answer |
| Currently lives in: | - countryside - country - city |
| Please indicate the number of days you have spent in social distancing to date. | Open answer |
| How many people besides you share the same domicile during the social distancing period? | Open answer |
| Does he/she work? | - Yes - No |
| Currently working: | - In smart work mode - At the place of work - I don't work due to the state of emergency |
| Compared to the two months preceding the social distancing period, how is your mood? | 1. Got worse 2. Unchanged 3. Improved |
| Due to the state of health emergency, please indicate how intensely you are feeling the following emotions:   - Anger - Disgust - Fear - Anxiety - Sadness - Happiness | 10-point scale (from 1= minimal intensity to 10= maximal intensity). |
| Were you infected with Covid-19? | - Yes - No - I don’t know |
| He/she has had/have symptoms: | - Yes - No |
| Currently: | - I'm cured - I'm in therapy |
| Do you think your headache puts you at greater risk of developing the disease? | - Yes - No - I don’t know |
| How afraid are you of manifesting the disease? | 10-point scale (from 1= minimal intensity to 10= maximal intensity). |
| Do you know people close to you who have been infected with Sars-Cov-2? | - Yes - No |
| Do you know people who died because of Sars-Cov-2? | - Yes - No |
| Compared to the two months preceding the social distancing / quarantine period, its feeding is: | - Reduced - Increased - It has not changed |
| Has your feeding changed in quality compared to the two months preceding the social distancing/quarantine period? | - Yes - No |
| Has your feeding changed during the hours compared to the two months preceding the social distancing / quarantine period? | - Yes - No |
| Compared to the two months preceding the social separation/quarantine period, its sleep quality considers it: | - worsened - same - improved |
| How many hours did you sleep per night in the two months preceding the social distancing/quarantine period? | Open answer |
| How many hours did you sleep on average each night during the social separation/quarantine period? | Open answer |
| Smoker? | - Yes - No |
| If you are a smoker, indicate the number of cigarettes consumed on average daily in the two months preceding the social distancing/quarantine period: | Open answer |
| If you are a smoker, indicate the number of cigarettes consumed on average daily during the social distancing/quarantine period: | Open answer |
| Do you take coffee? | - Yes - No |
| If yes, indicate the average number of coffees consumed daily in the two months preceding the social distancing/quarantine period: | Open answer |
| Indicate the average number of coffees consumed daily during the social distancing/quarantine period: | Open answer |
| Did you drink alcohol in the two months before the social distancing/quarantine period? | - Yes - No |
| How many times did you drink alcohol in the two months preceding the social distancing/quarantine period? | - Up to three times a week - More than three times a week - Everyday |
| During the period of social distancing/quarantine, are you consuming alcohol? | - Yes - No |
| If yes, indicate how many times a week you drink alcohol: | - Up to three times a week - More than three times a week - Everyday |
| On average, how many days per month of headache did you experience in the two months preceding the social distancing/ quarantine period? | Open answer |
| On average, how intense was the pain during the episodes of headache in the two months preceding the social distancing/quarantine period? | 10-point scale (from 1= minimal intensity to 10= maximal intensity). |
| How many days of headache have you had since the social distancing/quarantine period? | Open answer |
| On average, how intense was the pain during the episodes of headache you have had since the start of the social distancing/quarantine period? | 10-point scale (from 1= minimal intensity to 10= maximal intensity). |
| In the two months preceding the social distancing/quarantine period, was there a preventive therapy for migraine? | - Yes - No |
| If yes, indicate which drug you were taking: | Open answer |
| Did the preventive therapy continue a regular basis after the start of the period of social distancing/quarantine? | - Yes - No |
| If not, why has preventive therapy not been continued? | - Difficulty in finding the drug - Difficulty reaching the clinic for administration - I prefer to wait for the end of the social distancing/quarantine period - Other (specify) |
| On average, how many symptomatic medications did you take each month in the two months preceding the social distancing/ quarantine period? | Open answer |
| How many symptomatic medications have you taken since the start of the social distancing and quarantine period? | Open answer |
| How do you judge the progress of your headache since the beginning of the social distancing/quarantine period? | - Improved - Unchanged - Got worse |

|  |  |
| --- | --- |

**APPENDIX B**

| Domanda | Risposta |
| --- | --- |
| Genere | - Donna - Uomo |
| Eta’ (anni compiuti) | Risposta aperta |
| Anni di studio | - 5 - 8 - 13 - > 13 |
| Peso | Risposta aperta |
| Altezza | Risposta aperta |
| Regione di residenza | - Abruzzo - Basilicata - Calabria - Campania - Emilia-Romagna - Friuli-Venezia Giulia - Lazio - Liguria - Lombardia - Marche - Molise - Piemonte - Puglia - Sardegna - Sicilia - Toscana - Trentino-Alto Adige - Umbria - Valle d'Aosta - Veneto |
| In quale provincia abita attualmente? | Risposta aperta |
| In quale comune abita attualmente? | Risposta aperta |
| Attualmente, vive in: | - Campagna - Paese - Città |
| Indicare il numero di giorni che ha passato in distanziamento sociale fino ad oggi: | Risposta aperta |
| Quante persone oltre lei, condividono il suo stesso domicilio durante il periodo di distanziamento sociale? | Risposta aperta |
| Lavora? | - Si - No |
| Attualmente lavora: | - In modalità smart work - Presso sede di lavoro - Non lavoro a causa dello stato di emergenza |
| Rispetto ai due mesi precedenti al periodo di distanziamento sociale il suo umore è? | - Peggiorato - Invariato - Migliorato |
| A causa dello stato di emergenza sanitaria, indichi con quanta intensità sta provando le  seguenti emozioni:  Rabbia  Disgusto  Paura  Ansia  Tristezza  Felicità | Scala a 10 punti (da 1= minima intensità a 10= massima intensità) |
| E’ stato/a contagiato/a per Covid-19? | - Si - No - Non lo so |
| Ritiene che la sua cefalea la ponga a maggior rischio di sviluppare la malattia? | - Si - No - Non lo so |
| Quanto ha paura di manifestare la malattia? | Scala a 10 punti (da 1= minima intensità a 10= massima intensità) |
| Ha avuto/ha sintomi: | - Si - No |
| Attualmente: | - Sono guarito/a - Sono in terapia |
| Conosce persone a lei vicine che sono state contagiate dal Sars-Cov-2 (coronavirus)? | - Si - No |
| Conosce persone decedute a causa del SARS-CoV-2 (coronavirus)? | - Si - No |
| Rispetto ai due mesi precedenti al periodo di distanziamento sociale/quarantena la sua alimentazione è: | - Ridotta - Aumentata - Non è cambiata |
| Rispetto ai due mesi precedenti al periodo di distanziamento sociale/quarantena la sua alimentazione è cambiata nella qualità? | - Si - No |
| Rispetto ai due mesi precedenti al periodo di distanziamento sociale/quarantena la sua alimentazione è cambiata negli orari? | - Si - No |
| Rispetto ai due mesi precedenti al periodo di distanziamento sociale/quarantena ritiene che la qualità del sonno sia: | - Peggiorata - Uguale - Migliorata |
| Quante ore dormiva a notte nei due mesi precedenti al periodo di distanziamento sociale/quarantena? | Risposta aperta |
| Quante ore ha dormito in media ogni notte durante il periodo di distanziamento sociale/ quarantena? | Risposta aperta |
| Fumatore? | - Si - No |
| Se è un fumatore, indichi il numero di sigarette consumate in media  giornalmente nei due mesi precedenti al periodo di distanziamento sociale/quarantena: | Risposta aperta |
| Se è un fumatore indichi il numero di sigarette consumate in media giornalmente durante il periodo di distanziamento sociale/quarantena: | Risposta aperta |
| Assume caffè? | - Si - No |
| Se si, indichi il numero medio di caffè consumati giornalmente nei due mesi precedenti al periodo di distanziamento sociale/quarantena: | Risposta aperta |
| Indichi il numero medio di caffè consumati giornalmente durante il periodo di distanziamento sociale/quarantena: | Risposta aperta |
| Nei due mesi precedenti al periodo di distanziamento sociale/quarantena  consumava alcolici? | - Si - No |
| Quante volte consumava alcolici nei due mesi precedenti al periodo di distanziamento sociale/quarantena? | - Fino a 3 volte alla settimana - Più di 3 volte alla settimana - Tutti i giorni |
| Durante il periodo di distanziamento sociale/quarantena sta consumando  alcolici? | - Si - No |
| Se si, indichi quante volte a settimana assume alcolici: | - Fino a 3 volte alla settimana - Più di 3 volte alla settimana - Tutti i giorni |
| Durante i due mesi precedenti al periodo di distanziamento sociale/quarantena  praticava attività fisica? | - Si - No |
| Se si, quante volte a settimana praticava attività fisica: | - Fino a 3 volte alla settimana - Più di 3 volte alla settimana - Tutti i giorni |
| Durante il periodo di distanziamento sociale/quarantena sta svolgendo attività  fisica? | - Si - No |
| Se si, indichi quante volte alla settimana: | - Fino a 3 volte alla settimana - Più di 3 volte alla settimana - Tutti i giorni |
| In media, quanti giorni al mese di cefalea ha avuto nei due mesi precedenti il periodo di distanziamento sociale/quarantena? | Risposta aperta |
| In media, quanto è stata intensa la cefalea nei due mesi precedenti al periodo di distanziamento sociale/quarantena? | Scala a 10 punti (da 1= minima intensità a 10= massima intensità) |
| Quanti giorni di cefalea ha avuto dall'inizio del periodo di distanziamento sociale/quarantena? | Risposta aperta |
| In media, quanto è stato intenso il dolore durante gli episodi di cefalea che ha avuto dall'inizio del periodo di distanziamento sociale/quarantena? | Scala a 10 punti (da 1= minima intensità a 10= massima intensità) |
| Nei due mesi precedenti al periodo di distanziamento sociale/quarantena, era in  corso una terapia preventiva per l’emicrania? | - Si - No |
| Se si, indichi quale farmaco assumeva: | Risposta aperta |
| Dopo l’inizio del periodo di distanziamento sociale/quarantena la terapia preventiva è proseguita in modo regolare? | - Si - No |
| Se no, perché la terapia preventiva non è stata proseguita? | - Difficoltà nel reperimento del farmaco - Difficoltà a raggiungere l'ambulatorio per la somministrazione - Preferisco aspettare il termine del periodo di distanziamento sociale/quarantena - Altro (specificare) |
| In media, quanti sintomatici ha assunto ogni mese nei due mesi precedenti al periodo di distanziamento sociale/quarantena? | Risposta aperta |
| Quanti sintomatici ha assunto dall'inizio del periodo di distanziamento sociale/quarantena? | Risposta aperta |
| Come giudica l'andamento della sua cefalea dall'inizio del periodo di distanziamento sociale/quarantena? | - Migliorato - Invariato - Peggiorato |
