# Supplementary material for: Investigating the Effects of COVID-19 Quarantine in Migraine: An Observational Cross-Sectional Study From the Italian National Headache Registry (RICe)
Source: Front Neurol. 2020 Nov 10;11:597881. doi: 10.3389/fneur.2020.597881 (PMC7683429; doi:10.3389/fneur.2020.597881)
Supplement: Supplementary file 2 [file Table_1.DOCX]

**Supplemental Table 1**. Subjective perception of migraine in regard to CoViD-19.

|  | All (n=433) |
| --- | --- |
| Migraine perceived as a facilitating factor for CoViD-19, n (%) |  |
| Yes | 33 (7.6) |
| No | 347 (80.1) |
| Do not know | 53 (12.2) |
| Perceived outcome of migraine in relation to CoViD-19, n (%) |  |
| Improved | 145 (33.5) |
| Worsened | 103 (23.8) |
| Unchanged | 185 (42.7) |

**Supplemental Table 2**. Preventive treatment during social distancing compared with the previous two months in the included patients

|  | All (n=433) |
| --- | --- |
| Discontinued treatment, n (%) | 88 (20.3) |
| Reason for discontinuing, n (%) |  |
| Logistic* | 31 (35.2) |
| Drug failure** | 23 (26.1) |
| Other | 34 (38.6) |

*difficulties in drug administration (in-hospital treatments) or dispensing

**due to either ineffectiveness or adverse events

**S Table 3**. Baseline characteristics of the included subjects

|  | North (n=105) | Center (n=101) | South (n=227) | Statistic  Comparison | P value |
| --- | --- | --- | --- | --- | --- |
| Gender, n (%) |  |  |  | χ^2^ 3.7 | NS |
| Female | 77 (73.3) | 73 (72.3) | 183 (80.6) |  |  |
| Male | 28 (26.7) | 28 (27.7) | 44 (19.4) |  |  |
| Age (years), mean±SE | 47.24±1.17 | 42.00±1.51 | 43.36±0.71 | F 4.9 | 0.008* |
| BMI, mean±SE | 24±0.004 | 24±0.005 | 24±0.003 | F 0.12 | 0.880 |
| Headache frequency | 10.57±0.81 | 8.19±0.84 | 9.50±0.56 | F 2 | 0.12 |
| Education years, n (%) |  |  |  |  |  |
| 0-5 | 2 (1.9) | 4 (4.0) | 10 (4.4) |  |  |
| 6-8 | 29 (27.6) | 21 (20.8) | 34 (15.0) |  |  |
| 9-13 | 33 (31.4) | 52 (51.5) | 90 (39.6) |  |  |
| >13 | 41 (39.0) | 24 (23.8) | 93 (41.0) | χ^2^ 18.8 | 0.005 |
| Days of social distancing, mean±SE | 24.6±1.98 | 32.26±2.00 | 31.56±1.35 | F 4.9 | 0.008** |

BMI indicates body mass index; NS, not significant; SE, standard error

F: One way Anova test

χ^2 :^ Chi square

* (North vs Center) post hoc Bonferroni test

** (North vs Center vs South) post hoc Bonferroni test
